# Efficacy of Carbocisteine in Reducing Exacerbations in Chronic Obstructive Pulmonary Disease: A Systematic Review and Meta-Analysis of Randomized Controlled Trials

**DOI:** 10.3390/arm94010002

**Published:** 2025-12-31

**Authors:** Chia Siang Kow, Syed Shahzad Hasan, Kaeshaelya Thiruchelvam

**Affiliations:** 1 School of Pharmacy, Sunway University, Petaling Jaya 47500, Selangor, Malaysia; chiasiang_93@hotmail.com; 2 Sunway Biofunctional Molecules Discovery Centre, Faculty of Medical and Life Sciences, Sunway University, Petaling Jaya 47500, Selangor, Malaysia; s.hasan@hud.ac.uk; 3 School of Applied Sciences, University of Huddersfield, Huddersfield HD1 3DH, UK; 4 School of Pharmacy, IMU University, Bukit Jalil 57000, Kuala Lumpur, Malaysia

**Keywords:** carbocisteine, COPD, chronic obstructive pulmonary disorder, efficacy

## Abstract

**Highlights:**

**What are the main findings?**

- Carbocisteine lowered the rate of COPD exacerbations, supporting its use as an additional treatment to improve disease control.
- Carbocisteine was well tolerated, showing a safety profile similar to placebo with only mild side effects reported.

**What are the implications of the main findings?**

- Carbocisteine may be a useful add-on therapy for COPD patients who experience frequent exacerbations or have excess mucus, helping to improve symptom control and reduce flare-ups.
- Its good safety and low cost make carbocisteine a practical option, especially in resource-limited settings or for patients who cannot access or tolerate other advanced COPD treatments.

**Abstract:**

This systematic review and meta-analysis aimed to evaluate the efficacy and safety of carbocisteine in reducing chronic obstructive pulmonary disease (COPD) exacerbations based on evidence from randomized controlled trials (RCTs). A comprehensive literature search was conducted across PubMed, Embase, Cochrane Library, and ClinicalTrials.gov. RCTs comparing carbocisteine (1500 mg/day) with placebo in COPD patients, with a minimum follow-up of six months, were included. Data on exacerbation rates and adverse events were extracted and analyzed using a random-effects model. Four RCTs involving 1746 patients met inclusion criteria. Pooled analysis showed that carbocisteine significantly reduced the annual rate of acute exacerbations compared to placebo (WMD = −0.40; 95% CI: −0.69 to −0.11), with no significant increase in adverse events (OR = 1.02; 95% CI: 0.76 to 1.37). Mechanistically, carbocisteine improves mucociliary clearance, suppresses airway inflammation, reduces oxidative stress, and may hinder bacterial colonization. Carbocisteine is associated with a significant reduction in COPD exacerbations and demonstrates a favorable safety profile. It may serve as an effective adjunctive therapy in patients with frequent exacerbations and mucus hypersecretion.

## 1. Introduction

Chronic obstructive pulmonary disease (COPD) is a chronic, progressive respiratory disorder characterized by persistent airflow limitation, airway inflammation, and recurrent exacerbations [1]. Exacerbations play a critical role in disease progression, leading to worsened lung function, increased symptom burden, reduced quality of life, and heightened healthcare utilization. Repeated exacerbations not only accelerate lung function decline but are also associated with higher morbidity and mortality [2], emphasizing the urgent need for effective pharmacological strategies to prevent exacerbations and improve long-term COPD management.

Mucolytic agents, such as carbocisteine, have been widely explored as potential adjunctive therapies for COPD, particularly in patients with chronic mucus hypersecretion [3]. Carbocisteine is a thiol-based mucoregulator that acts by modulating mucus production, reducing its viscosity, and enhancing mucociliary clearance, thereby preventing mucus accumulation and airway obstruction. In addition to its mucoregulatory effects, carbocisteine has been shown to possess anti-inflammatory and antioxidant properties, which may contribute to reducing airway inflammation and oxidative stress, both of which are key drivers of COPD exacerbations [4]. By mitigating these pathological processes, carbocisteine may play a role in reducing exacerbation frequency.

Despite its proposed benefits, the efficacy of carbocisteine in COPD remains controversial, with studies reporting variable outcomes. While some clinical trials suggest that carbocisteine significantly reduces the frequency of exacerbations and improves overall respiratory health, others have found no substantial benefit compared to placebo. These conflicting findings highlight the need for a comprehensive synthesis of available evidence to determine the true clinical impact of carbocisteine in COPD management.

Although a comprehensive Cochrane review [5] has previously assessed mucolytics for COPD, its conclusions were derived from trials evaluating a broad range of pharmacologically diverse agents, including N-acetylcysteine, erdosteine, ambroxol, and several others. As a result, the overall estimates reflect class-level effects rather than agent-specific performance. Carbocisteine, however, is prescribed as a distinct therapeutic entity in routine practice, and its clinical relevance is supported by widespread use in Europe and Asia as well as by mechanistic actions—such as modulation of mucin expression, enhancement of mucociliary clearance, and reduction of oxidative stress—that are not uniformly shared across the mucolytic class. Since the Cochrane review, a new multicentre randomized controlled trial of carbocisteine has been published in 2025, providing contemporary data that have not been synthesized within existing reviews. These considerations highlight the need for a focused, updated meta-analysis that evaluates carbocisteine independently, allowing clinicians to better interpret the magnitude and consistency of its therapeutic benefit based on drug-specific evidence.

This systematic review and meta-analysis aimed to provide a rigorous, evidence-based evaluation of carbocisteine’s effect on COPD exacerbations and safety profile by analyzing data from randomized controlled trials (RCTs).

## 2. Materials and Methods

A comprehensive literature search was performed using PubMed, Embase, Cochrane Library, and ClinicalTrials.gov from inception to the latest available date. Keywords included “carbocisteine,” “chronic obstructive pulmonary disease,” “COPD exacerbations,” and “randomized controlled trial” with appropriate Boolean operators. Reference lists of relevant studies and systematic reviews were manually screened for additional eligible trials.

The full electronic search strategies used for each database were as follows:

- PubMed:
  (“carbocisteine”[Mesh] OR “carbocisteine”[tiab] OR “S-carboxymethylcysteine”[tiab])
  AND (“chronic obstructive pulmonary disease”[Mesh] OR “COPD”[tiab] OR “chronic bronchitis”[tiab])
  AND (“exacerbation*”[tiab] OR “acute exacerbation*”[tiab])
  AND (“randomized controlled trial”[pt] OR “randomized”[tiab] OR “placebo”[tiab])
- Embase:
  (‘carbocisteine’/exp OR ‘carbocisteine’:ti,ab)
  AND (‘chronic obstructive lung disease’/exp OR ‘COPD’:ti,ab OR ‘chronic bronchitis’:ti,ab)
  AND (‘exacerbation’:ti,ab OR ‘acute exacerbation’:ti,ab)
  AND (‘randomized controlled trial’/exp OR ‘randomized’:ti,ab OR ‘placebo’:ti,ab)
- Cochrane Library:
  carbocisteine:ti,ab,kw
  AND (COPD OR “chronic obstructive pulmonary disease” OR “chronic bronchitis”)
  AND (exacerbation OR “acute exacerbation”)
  Filter: Trials
- ClinicalTrials.gov:
  Condition: “COPD” OR “chronic obstructive pulmonary disease”
  Intervention: “carbocisteine”
  Study type: Interventional
  Status: Completed

Studies were included if they were randomized, double-blind, placebo-controlled trials evaluating carbocisteine in patients with COPD or chronic obstructive bronchitis diagnosed using contemporaneous guideline-based and spirometry-supported criteria, as reported in each trial. Across studies, diagnosis was based on established clinical definitions of chronic bronchitis or COPD, objective evidence of persistent airflow limitation (e.g., FEV_1_/FVC < 0.70 or FEV_1_ 40–79% predicted), and exclusion of alternative respiratory diagnoses, consistent with internationally accepted standards and later Global Initiative for Chronic Obstructive Lung Disease (GOLD) diagnostic principles [6]. The intervention group received carbocisteine (1500 mg/day), while the comparator group received a placebo. Eligible studies reported at least one of the following outcomes: rate of acute COPD exacerbations and adverse events. To ensure clinical relevance, only studies with a minimum follow-up duration of six months were included.

Studies were excluded if they lacked a placebo-controlled design, were observational studies, case reports, or non-randomized trials, or did not report relevant outcome data. The study selection process was conducted independently by two authors (CSK and SSH), who screened the titles and abstracts of all identified records. Full-text articles of potentially eligible studies were retrieved and assessed against the inclusion and exclusion criteria. Disagreements were resolved through discussion and consensus, and a third reviewer (KT) was consulted if needed. The final selection of studies was determined after reaching agreement among all reviewers.

Two independent reviewers (CSK and SSH) extracted data using a standardized form, capturing key study characteristics such as location, study design, and duration, as well as patient demographics, including sample size and mean age. Data on exacerbation rates (mean rate per year for carbocisteine vs. placebo) and adverse events (number of reported events in each group) were also collected.

The Cochrane Risk of Bias 2 (RoB2) tool [7] was used to assess the quality of included trials, evaluating five domains: randomization process, deviation from intended interventions, missing outcome data, measurement of the outcome, and selection of reported results. The overall risk of bias for each study was categorized as low risk (all domains judged as low risk), some concerns (at least one domain raised concerns but no high-risk domains), or high risk (at least one domain was high risk).

A meta-analysis was performed using MetaXL (EpiGear International Pty Ltd., Sunshine Coast, Australia, version 5.3). The effect of carbocisteine on exacerbation rates was reported as weighted mean difference (WMD) with 95% confidence intervals (CI) using a random-effects model, while adverse events were analyzed using pooled odds ratios (OR) with 95% CI, calculated with the Mantel–Haenszel method under a random-effects model. A random-effects model was applied to account for expected heterogeneity due to differences in study populations, study durations, baseline exacerbation rates, and geographic variations in COPD management. Statistical heterogeneity was assessed using Cochran’s Q test and I^2^ statistic. A *p*-value < 0.10 in Cochran’s Q test indicated significant heterogeneity, while I^2^ > 50% suggested substantial variability among studies.

## 3. Results

A total of 65 records were identified through database searches and manual reference screening. After removing 34 duplicates, 31 articles remained for title and abstract screening. Following full-text assessment, four studies [8,9,10,11] were included in the systematic review and meta-analysis, comprising 1746 patients with COPD. All included trials were randomized, double-blinded, placebo-controlled and had a study duration ranging from 6 to 12 months. The dose of carbocisteine was consistently 1500 mg/day across studies. Patient demographics were similar, with a mean age ranging from 60.0 to 72.7 years. Table 1 provides an overview of the key characteristics of the included studies.

All four trials [6,7,8,9] were assessed using the Cochrane Risk of Bias 2 tool. Each study demonstrated a low risk of bias across all major domains, including the randomisation process, deviations from intended interventions, missing outcome data, measurement of outcomes, and selection of reported results. The overall quality of the included evidence was therefore considered robust, with no methodological concerns that would materially affect the validity of the pooled estimates.

Across the four included randomized controlled trials [8,9,10,11], acute exacerbations were defined using broadly similar but study-specific clinical criteria reflecting internationally accepted standards. In the multicentre Italian trial by Allegra et al. [8], exacerbations were identified based on predefined clinical worsening recorded during follow-up, although a detailed operational definition was not explicitly stated in the published report. The Japanese trial by Yasuda et al. [9] defined a COPD exacerbation as “an acute and sustained worsening of COPD symptoms requiring changes to regular treatment”, consistent with commonly used clinical practice criteria. The large Chinese PEACE study by Zheng et al. [10] applied the Anthonisen criteria, defining an exacerbation as “at least two days of persistence of two major symptoms (worsening dyspnoea and increased sputum volume or purulence) or one major symptom plus more than one minor symptom (upper airway infection, unexplained fever, or increased wheezing)”. The recent mild-to-moderate COPD trial by Zhou et al. [11] similarly defined exacerbations as “worsening of at least two major symptoms (cough, sputum volume, sputum purulence, wheezing, or dyspnea) lasting at least 48 h, after excluding alternative cardiopulmonary causes”. Taken together, all trials employed definitions centred on acute symptomatic deterioration, with the more recent large-scale studies utilising explicit, internationally validated criteria that align closely with GOLD and Anthonisen standards.

**Figure 1:**
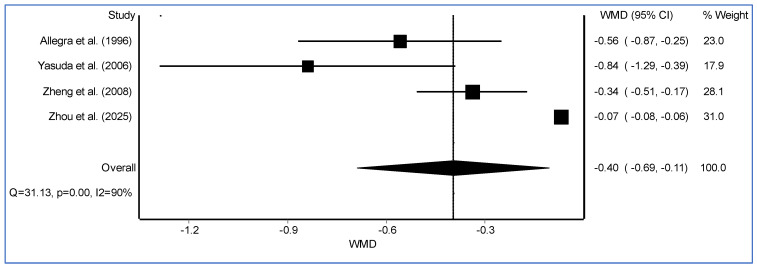
The forest plot illustrates the weighted mean difference (WMD) with 95% confidence intervals (CI) for individual randomized controlled trials assessing the effect of carbocysteine versus placebo on COPD exacerbation rates [8,9,10,11].

Pooled analysis showed that carbocisteine significantly reduced the mean annual rate of acute COPD exacerbations compared to placebo (WMD = −0.40, 95% CI: −0.69 to −0.11, I^2^ = 90%, Figure 1). This supports its role as an adjunctive therapy in COPD management. The beneficial effects of carbocisteine are attributed to its mucoregulatory, antioxidant, and anti-inflammatory properties, which collectively improve airway clearance and reduce exacerbation frequency. Pooled analysis of adverse events showed no significant difference between carbocisteine and placebo (OR = 1.02, 95% CI: 0.76 to 1.37, I^2^ = 0%), suggesting a favorable safety profile.

## 4. Discussion

A key mechanism by which carbocisteine exerts its effects is through mucoregulation and airway clearance [4]. COPD is characterized by excessive mucus production and impaired mucociliary function, leading to airway obstruction and an increased risk of infections. Carbocisteine has been shown to reduce goblet cell hyperplasia and inhibit mucin (MUC5AC and MUC5B) overexpression [12,13], which contribute to mucus hypersecretion. Additionally, by enhancing mucociliary transport, carbocisteine facilitates mucus clearance, reducing mucus plugging—a key factor in exacerbation episodes [14]. These effects may help prevent bacterial colonization, which frequently triggers COPD exacerbations.

Beyond mucoregulation, carbocisteine exhibits antioxidant properties that may play a crucial role in reducing COPD exacerbations [15]. Oxidative stress is a major contributor to COPD progression, leading to inflammation, airway remodeling, and recurrent exacerbations. Carbocisteine increases glutathione (GSH) levels, a key antioxidant that neutralizes reactive oxygen species (ROS), thereby protecting lung tissue from oxidative damage [16]. By mitigating oxidative stress, carbocisteine may help prevent airway injury and inflammation, reducing the frequency and severity of exacerbations.

Carbocisteine also possesses anti-inflammatory effects, which may contribute to its therapeutic benefit in COPD [17]. Chronic inflammation in COPD involves increased activity of neutrophils, macrophages, and pro-inflammatory cytokines (e.g., IL-6, IL-8, TNF-α), all of which promote airway destruction and disease progression. Carbocisteine has been shown to suppress pro-inflammatory cytokines and inhibit neutrophil activation, leading to a reduction in airway inflammation. Additionally, by modulating the nuclear factor kappa B (NF-κB) signaling pathway, carbocisteine further dampens the inflammatory cascade, helping to maintain airway stability and reduce exacerbation risk [18].

Another potential mechanism of carbocisteine is its effect on bacterial adhesion and biofilm formation [15]. COPD exacerbations are frequently triggered by bacterial infections, particularly by *Haemophilus influenzae*, *Streptococcus pneumoniae*, and *Moraxella catarrhalis* [19]. Studies suggest that carbocisteine may reduce bacterial adhesion to airway epithelial cells, limiting colonization and infection. Additionally, carbocisteine has been reported to interfere with biofilm formation, which plays a key role in chronic bacterial persistence within the airways. By reducing bacterial load, carbocisteine may decrease the likelihood of infection-driven exacerbations.

Pooled analysis of adverse events showed no significant difference between carbocisteine and placebo, suggesting a favorable safety profile. The most commonly reported adverse events included gastrointestinal discomfort and mild respiratory symptoms, but these were not significantly different from placebo, indicating that carbocisteine is well-tolerated in COPD patients. Gastrointestinal symptoms—particularly dyspepsia, nausea, diarrhoea, and abdominal discomfort—were the most frequently reported events and occurred in both carbocisteine and placebo groups. Mild upper respiratory symptoms such as sore throat, cough, rhinorrhoea, and common-cold–like complaints were also described, reflecting background respiratory illness rather than drug-specific effects. Several studies reported sporadic headaches, dizziness, or minor skin reactions, while serious adverse events were uncommon and typically unrelated to treatment. Importantly, none of the trials identified meaningful safety signals involving hepatotoxicity, nephrotoxicity, or cardiovascular complications. Taken together, the evidence indicates that carbocisteine is well tolerated in COPD populations, with an adverse-event profile comparable to placebo and no indication of clinically significant harm during 6–12 months of therapy.

Given these findings, carbocisteine may be considered as an adjunctive therapy in COPD management, particularly for patients with frequent exacerbations, mucus hypersecretion, or those not receiving inhaled corticosteroids (ICS). The low cost and favorable safety profile of carbocisteine further support its potential utility, especially in settings where access to advanced COPD treatments is limited.

However, several limitations must be acknowledged. The moderate heterogeneity among included studies suggests variability in patient populations, study durations, and disease severity, which may influence the generalizability of results. Additionally, most included trials had a maximum follow-up of 12 months, limiting insights into the long-term effects of carbocisteine on disease progression, hospitalizations, and mortality. Future research should focus on long-term clinical trials and real-world studies to better establish the role of carbocisteine in COPD management. Furthermore, the small number of eligible randomized trials precluded any meaningful assessment of publication bias using conventional statistical approaches such as funnel plot asymmetry or Egger’s test. While the direction and consistency of effect estimates across studies are reassuring, the possibility of unpublished negative or neutral trials cannot be fully excluded.

## 5. Conclusions

In conclusion, this meta-analysis provides suggestive evidence that carbocisteine significantly reduces COPD exacerbations, likely due to its mucoregulatory, antioxidant, and anti-inflammatory properties. Given its favorable safety profile and potential clinical benefits, carbocisteine may serve as an effective adjunct therapy in COPD patients with frequent exacerbations and mucus hypersecretion. Further studies are needed to determine its long-term impact on disease progression and overall COPD outcomes.

## Figures and Tables

**Table 1:** Characteristics of included trials.

Study (Year)	Location	Study Design	Duration of Study (Months)	Dose of Carbocisteine (mg/Day)	Number of Patients (Treatment–Control)	Mean Age (SD), Years	Mean Annual Rate of Acute COPD Exacerbation (SD)		Adverse Events (n/N; %)	
							Carbocisteine	Placebo	Carbocisteine	Placebo
**Allegra et al. (1996)** [8]	Italy	Randomized, double-blinded, controlled trial	6	1500	171:181	60.0 (9.8)	0.92 (1.46)	1.48 (1.48)	6/171; 3.5	11/181; 6.1
**Yasuda et al. (2006)** [9]	Japan	Randomized, double-blinded, controlled trial	12	1500	78:78	72.7 (8.8)	0.54 (0.97)	1.38 (1.77)	N/A	N/A
**Zheng et al. (2008)** [10]	China	Randomized, double-blinded, controlled trial	12	1500	353:354	65.2 (9.2)	1.01 (1.13)	1.35 (1.13)	57/353; 16.2	56/354; 15.8
**Zhou et al. (2025)** [11]	China	Randomized, double-blinded, controlled trial	12	1500	356:175	64 (7)	0.39 (0.03)	0.46 (0.05)	65/362; 18.0	28/177; 15.8

N/A: Not Applicable.

## Data Availability

Data will be made available upon reasonable request.

## Abbreviations

The following abbreviations are used in this manuscript:

COPD	Chronic obstructive pulmonary disease
GOLD	Global Initiative for Chronic Obstructive Lung Disease
NF-κB	Nuclear factor kappa B
RCT	Randomized controlled trial
